# Empiric isolation of the superior vena cava in atrial fibrillation patients: old concept, new insights?

**DOI:** 10.1093/europace/euae041

**Published:** 2024-02-02

**Authors:** Fabian Moser, Andreas Rillig, Andreas Metzner

**Affiliations:** Department of Cardiology, University Heart and Vascular Center Hamburg-Eppendorf, Martinistr. 52, 20246 Hamburg, Germany; Department of Cardiology, University Heart and Vascular Center Hamburg-Eppendorf, Martinistr. 52, 20246 Hamburg, Germany; Department of Cardiology, University Heart and Vascular Center Hamburg-Eppendorf, Martinistr. 52, 20246 Hamburg, Germany

**This editorial refers to ‘Role of electroanatomical mapping–guided superior vena cava isolation in paroxysmal atrial fibrillation patients without provoked superior vena cava trigger: a randomized controlled study’ by Y. Dong *et al*., https://doi.org/10.1093/europace/euae039.**

Pulmonary veins (PVs) are the dominant sources of ectopies that initiate and perpetuate atrial fibrillation (AF). Electrical isolation of the PVs is therefore the cornerstone of all AF ablation strategies and durable PV isolation (PVI) is crucial for clinical outcome after catheter ablation.^1^ However, non-PV trigger initiating AF was reported to occur in 20–30% of patients.^2^ These triggers can arise from different left atrial and right atrial (RA) areas and structures. The superior vena cava (SVC) is discussed to be one of the common sources of ectopies that might initiate AF.^3,4^

The study by Dong *et al*. investigated the characteristics of SVC-triggered AF and the role of electroanatomical mapping–guided SVC isolation in addition to PVI on the outcome of catheter ablation in patients with paroxysmal AF in the absence of documented SVC triggers. In the study cohort in nearly 77% of patients with paroxysmal AF, no SVC triggers could be identified. Patients without identifiable SVC triggers were randomized to PVI plus SVC isolation vs. PVI only to compare the 12-month success rate. Superior vena cava isolation in addition to PVI did not improve freedom from atrial tachyarrhythmias in this patient cohort.^5^

There are several reports about the extension of RA muscular sleeves into the SVC and their excitability. These myocardial extensions were found to be present in nearly 80% of human hearts in a postmortem study.^6^ Heterogeneous fibre arrangements of myocardial sleeves penetrating fibrous tissue could potentially form the substrate for heterogeneity of electrical coupling leading to anisotropic conduction and arrhythmogenicity. Autonomic influences can promote spontaneous automaticity and triggered activity in the SVC sleeves.^7^

Conflicting data exist on whether isolation of SVC triggers can improve long-term maintenance of sinus rhythm after AF ablation.^4,8^ Two different strategies for SVC isolation have been described. Either SVC isolation is only performed when triggers from the SVC can be documented during the procedure or SVC isolation is performed empirically and independently of SVC triggers in addition to PVI.^9^

The current data by Dong *et al*. suggest no benefit when SVC isolation is performed empirically. In order to avoid unnecessary ablation and potential complications, SVC isolation should therefore only be considered in patients who present with AF and recorded SVC-related triggers. Durable PVI should be prerequisite for considering any additional ablation of areas with non-PV triggers in patients with AF.

##  

### Procedural aspects of superior vena cava isolation

Initially, the arrhythmogenic foci inside the SVC were mapped and focal ablation of the foci in the SVC was performed.^10^ However, since arrhythmogenicity can be multifocal and ablation within the SVC can cause potential complications, the current standard is the electrical isolation of the SVC at the SVC–RA junction. The electric junction can be defined by the presence of merged SVC and local RA potentials recorded by a circular mapping catheter. The precise demarcation using angiography, magnetic resonance imaging or computed tomography scan, and/or a three-dimensional electroanatomical mapping system is essential. Isoproterenol infusion, pacing manoeuvres, and/or cardioversion might be needed to provoke and record SVC triggers. Superior vena cava isolation can be achieved by delivering sequential RF applications at the site of earliest SVC activation (breakthrough) during sinus rhythm. Radiofrequency (RF) energy is usually applied from 20 W and can be titrated up to 30 W. The endpoint is the elimination or dissociation of all SVC potentials.^11^

Alternatively, SVC isolation was also induced by cryoballoon ablation in a prospective series of patients. However, caution must be taken, as the complication rate was reported to be considerably high.^12^

Pulsed field ablation (PFA) as a non-thermal energy source in which electrical fields are used to disrupt the membrane of cardiomyocytes might be a novel alternative since it reduces potential damage to extracardiac anatomical structures, such as the adjacent right phrenic nerve. Currently, there are only single case reports on PFA to target the SVC.^13^ In a previous report, one patient experiencing recurrent atrial fibrillation (AF) episodes following prior PFA-based PVI, superior vena cava (SVC) triggers were identified and the patient underwent PFA-based ablation within the SVC.^14^ The PFA catheter was positioned within the SVC and four PFA applications were set. During these applications, sinus arrest occurred and, after 5 min of atrial pacing, sinus rhythm returned spontaneously and AF did not reoccur during follow-up (*Figure 1*). However, more studies are needed before final conclusions can be drawn regarding the potential role of PFA for SVC ablation.

**Figure 1:**
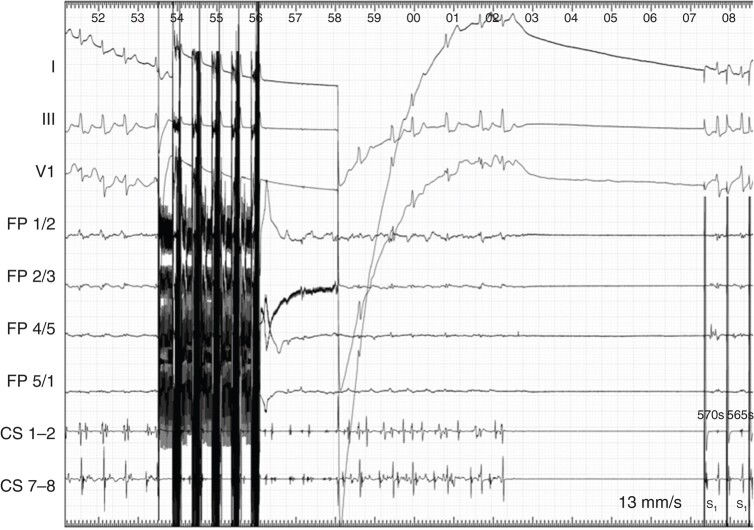
Example of transient sinus node arrest after a pulsed field application at the superior vena cava. Atrial fibrillation terminates with transient sinus arrest. Atrial pacing was performed for 5 min before sinus rhythm > 40 b.p.m. recovered.

### Potential complications of superior vena cava isolation

Superior vena cava stenosis can occur after extensive RF ablation. Callans *et al*. reported SVC narrowing after multiple RF applications at the SVC–RA junction in patients with inappropriate sinus tachycardia. In this study, local and circumferential swelling of the SVC was observed, causing a progressive reduction of the SVC diameter by 24%.^15^ There is also a case report of severe stenosis of the SVC after RF ablation to obtain SVC isolation using an irrigated-tip catheter (25 W, 10 times, 278 s).^16^

In contrast to antral PVI, which is the current standard to prevent the risk of PV stenosis, antral ablation of the SVC bears the risk of sinus node injury. Therefore, SVC isolation should be performed at the ‘electrical junction’, defined by the presence of merged SVC and local RA potentials, ∼5–10 mm above the anatomical SVC–RA junction.^17^ Radiofrequency application should be stopped immediately if an acceleration of sinus rhythm is observed.

Phrenic nerve injury is another potential complication. Damage to the phrenic nerve mostly occurs during RF applications at the posterolateral aspect of the SVC or during cryoballon ablation of the SVC. Pacing manoeuvre with high output to confirm non-capture of the right phrenic nerve should be performed before and during ablation to avoid phrenic nerve injury. Vigilant monitoring of phrenic nerve function during CB ablation of SVC is mandatory.

## Data Availability

All relevant data are within the manuscript.
